# The association between fear of COVID‐19 and mental health: The mediating roles of burnout and job stress among emergency nursing staff

**DOI:** 10.1002/nop2.1154

**Published:** 2021-12-08

**Authors:** Daniel Kwasi Ahorsu, Chung‐Ying Lin, Zohreh Hosseini Marznaki, Amir H. Pakpour

**Affiliations:** ^1^ Department of Rehabilitation Sciences Faculty of Health & Social Sciences The Hong Kong Polytechnic University Kowloon Hong Kong; ^2^ Institute of Allied Health Sciences College of Medicine National Cheng Kung University Tainan Taiwan; ^3^ Department of Nursing Amol Faculty of Nursing and Midwifery Mazandaran University of Medical Sciences Sari Iran; ^4^ Social Determinants of Health Research Center Research Institute for Prevention of Non‐Communicable Diseases Qazvin University of Medical Sciences Qazvin Iran; ^5^ Department of Nursing School of Health and Welfare Jönköping University Jönköping Sweden

**Keywords:** burnout, fear of COVID‐19, job stress, mediation, mental health, nurse

## Abstract

**Aim:**

This current study examined the mediation roles of burnout and job stress in the association between fear of COVID‐19 and mental health among emergency nurses.

**Design:**

Cross‐sectional survey.

**Methods:**

A total of 516 emergency nurses with a mean age of 41.18 (*SD* = 8.24), mostly females (76.16%) and married (78.9%) responded to measures on fear of COVID‐19, burnout, job stress and mental health between 15 September and 7 November 2020.

**Results:**

There was a significantly direct effect between (a) fear of COVID‐19 and mental health, (b) fear of COVID‐19 and mediators (burnout and job stress) and (c) mediators (burnout and job stress) and mental health. Also, there was a significantly indirect effect between fear of COVID‐19 and mental health through job stress, burnout or both.

**Conclusions:**

Fear of COVID‐19 directly and indirectly influenced the mental health of emergency nurses. Therefore, hospital authorities should provide sufficient resources to allay the fears of nurses during this COVID‐19 pandemic period.

## 1 INTRODUCTION

The emergence of COVID‐19 and its enervating effects on lifestyle (Balanzá–Martínez et al., 2020; Lippi et al., 2020; Van Lancker & Parolin, 2020), health (Ahorsu, Imani, et al., 2020; Ahorsu, Lin & Pakpour et al., 2020; Fazeli et al., 2020) and economies around the world (Gössling et al., 2020; Nicola et al., 2020) are very disturbing. With the increasing COVID‐19 cases (over 234 million), severities and fatalities (over 4.8 million) worldwide as of 5th October 2021 (World Health Organisation, 2021), emergency department (especially if they oversee the treatment of COVID‐19 cases) staff members may get overwhelmed by the influx of COVID‐19 cases which directly increases their workload. This is especially a challenging issue among emergency nurses as they have to triage all patients including COVID‐19 cases within a limited time period. This consequently increases their job stress as they have to deal with triaging and treating different cases with limited personal protective equipment (PPEs) and within a limited time period (Al Thobaity & Alshammari, 2020; Shoja et al., 2020; Wang et al., 2020).

## 2 BACKGROUND

Recent studies have indicated that frontline workers which include emergency nurses have increased job stress as they have added tasks and new protocols which must be strictly adhered to due to the COVID‐19 pandemic (Al Thobaity & Alshammari, 2020; Shoja et al., 2020; Wang et al., 2020). Other studies which reported reduced non‐COVID‐19 case visits to the emergency department indicated that the workload remained unchanged or increased due to changes and special extra tasks that needs to be performed by the emergency nurses (Shoja et al., 2020; Sung et al., 2020; Zhan et al., 2020). An increased workload/job stress may have cascading adverse effects on relationships, appropriate service delivery and overall job performance among nurses (Cui et al., 2021; Gualano et al., 2021; Zare et al., 2021). The situation may even be worse when most nurses fear COVID‐19 due to the limited PPEs, and other uncertainties surrounding COVID‐19, and unavailability of a cure or definite treatment (Al Thobaity & Alshammari, 2020; Çınar et al., 2021; Zare et al., 2021).

One of the most challenging aspects of COVID‐19 is the indefinite transmission medium which includes but not limited to respiratory droplets during close face‐to‐face spread by asymptomatic, presymptomatic and symptomatic carriers (Wiersinga et al., 2020). This situation is especially challenging for the emergency staff members because they are charged with overseeing the treatment of COVID‐19 cases and more specifically emergency nurses as they have to attend to trauma‐related and other life‐death cases that have unknown COVID‐19 status. This increases their chance of contracting COVID‐19 and hence increasing their fear of COVID‐19. The fear of COVID‐19 combined with limited PPEs and inadequate information among the nurses may hinder effective delivery of quality services to patients as the nurses may have decreased job satisfaction, increased psychological distress, performance, and increased organizational and professional turnover intentions (Al Thobaity & Alshammari, 2020; De los Santos & Labrague, 2021; Labrague & de Los Santos, 2021; Rajabimajd et al., 2021; Shoja et al., 2020; Yayla & Eskici İlgin, 2021). Apart from the poor delivery of quality services, job stress in tandem with fear of contracting COVID‐19 may cause burnout and mental health challenges among the nurses (Hu et al., 2020; Zare et al., 2021).

Burnout, syndrome of three feelings (emotional exhaustion, depersonalization and low personal accomplishment; Maslach et al., 2018), among nurses is mostly due to factors such as excessive workload, low rewards and poor relationships with colleagues (Dall'Ora et al., 2020) which unfortunately may further lead to other mental health challenges if not properly dealt with (Hu et al., 2020; Shoja et al., 2020). That is, with the increasing COVID‐19 cases in the hospital, inadequate resources and support structures to cope with the influx of cases, nurses may be prone to burnout (physical and emotional exhaustion, depersonalization, and low personal accomplishment). The continuous burnout among nurses may deteriorate their health and specifically their mental health which may affect their performance at work (Hamed et al., 2020; Zeng et al., 2020). That is, burnout has strongly been associated with reduced job performance, poor quality of care, poor patient safety, adverse events, patient negative experience, medical errors, infections, patient falls and intention to leave (Al Sabei et al., 2020; Dall'Ora et al., 2020; Kwon et al., 2021) This leaves a huge financial burden and serious administrative challenges for the health authorities (managers and directors). For instance, the estimated financial cost for burnout‐related turnover among US physicians and nurses was $17 and $14 billion, respectively, per annum (National Taskforce for Humanity in Healthcare, 2018). In other reports, the financial burden of burnour and/or mental health among nurses or healthcare workers are in several billions dollars per anum (Kline & Lewis, 2019; Medibank, 2011; Perry et al., 2015; Torjesen, 2018). Yet, no known study has examined the mediating roles of burnout and job stress in the association between fear of COVID‐19 and mental health.

### 2.1 Research question

**FIGURE 1:**
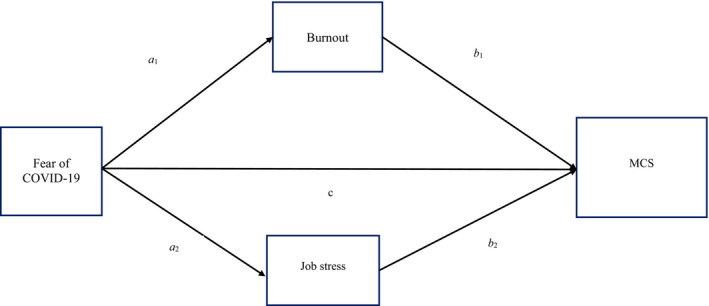
Mediation model with burnout and Job stress as proposed mediators of the effect of fear of COVID‐19 on emergency nursing staff's mental health. Note: Age, sex, years of experience and marital status were adjusted for the model. MCS, mental component summary

Hence, and considering current COVID‐19 situation, this current study aimed to examine the mediation roles of burnout and job stress in the association between fear of COVID‐19 and mental health (see Figure 1). The findings will be beneficial to hospital authorities and health professionals themselves by providing information on how to handle their workplace challenges and their mental health appropriately. It will also serve as a background for future clinical studies among health professionals during this COVID‐19 pandemic period.

## 3 THE STUDY

### 3.1 Design

This study used the cross‐sectional design and with the help of a survey to gather the needed data from emergency nursing staff.

### 3.2 Method

#### 3.2.1 Participants and procedure

This study recruited 516 emergency nursing staff between 15 September and 7 November 2020 from 18 hospitals in Qazvin (Iran) using a list of nursing staffs who were working at emergency departments as prepared by Treatment Affairs (Qazvin, Iran). An online questionnaire was sent by SMS to 800 staff members and 516 (response rate of 64.5%) of them agreed to participate in the study. Inclusion criteria were as follows: educated in nursing, working at emergency department and provided online consent form for participation.

#### 3.2.2 Measures

##### Fear of COVID‐19

The emergency nurses' fear of COIVD‐19 was assessed using the fear of COVID‐19 scale. This scale was developed by Ahorsu, Lin, Imani, et al. (2020). It is made up of seven items which are rated on a five‐point Likert‐type scale response format (strongly agree = 5 to strongly disagree = 1). The total score which was generated by adding all the responses ranged from 7 to 35. Hence, higher scores indicated greater fear of COVID‐19. The Persian version was used for this study with an acceptable internal consistency (*α* = 0.82; Ahorsu, Lin, Imani, et al., 2020).

##### Job stress

The emergency nurses' occupational stress was assessed using the occupational stress inventory (revised) which was developed by Osipow (1998). It is made up of six subscales (role overload, role insufficiency, role ambiguity, role boundary, responsibility and physical environment) with 10 items per each subscale. It is rated on a five‐point Likert‐type scale response format (1 = never, 2 = occasionally, 3 = sometimes, 4 = usually and 5 = most of the time) with a higher subscale score depicting a greater level of role stressor. The Persian version was used for this study with an acceptable internal consistency (*α* = 0.83; Sharifian et al., 2006).

##### Burnout

The nurses' burnout was assessed using the Maslach Burnout Inventory Human Services Survey for Medical Professionals, fourth edition (MBI‐HSS MP, 4th Ed.) developed by Maslach et al. (2018). It is made up of 22 items with frequency rating choices of Never (0), A few times a year or less (1), Once a month or less (2), A few times a month (3), Once a week (4), A few times a week (5) or Every day (6) that covers 3 areas (Emotional Exhaustion, EE; Depersonalization, DP; and low sense of Personal Accomplishment, PA). Hence, three MBI scale scores were calculated and interpreted separately for each nurse. The Persian version of MBI showed acceptable Cronbach's alpha coefficients (0.71–0.85) from a previous study Moalemi et al. (2018). Moreover, the Cronbach's alpha coefficients of MBI calculated using the present study's data were acceptable with the range between 0.73 and 0.83.

##### Mental health

The nurses' mental health was assessed using the 12‐Item Short Form Health Survey (SF‐12) developed by Ware Jr et al. (1996). Specifically, this study used the Mental Component Summary (MCS) of the scale. The Persian version was used for this study with acceptable Cronbach's alpha coefficient of above 0.82 (Montazeri et al., 2011; Pakpour et al., 2011).

### 3.3 Data analysis

The demographic information of the participants was presented using descriptive statistics, including mean (*SD*) and frequency (percentage). Then, Pearson's correlation was applied to examine the bivariate correlations among the studied variables. After the confirmation of the associations (as shown by the Pearson correlation) among the studied variables, structural equation modelling using full information maximum likelihood estimator was used to examine the proposed mediation model (Figure 1). More specifically, fear of COVID‐19 was the independent variable, job stress and burnout were the mediators, and mental health was the outcome variable. Age, sex, years of clinical experience and marital status were adjusted for the mediation model. Moreover, bootstrapping method with a total of 5,000 resamples was used to examine the mediation effect.

Several fit indices, including comparative fit index (CFI) (Bentler, 1990), Tucker–Lewis index (TLI) (Tucker & Lewis, 1973), standardized root mean square residual (SRMR) and root mean square error of approximation (RMSEA) (Joreskog & Sorbom, 1981), were used to examine whether the mediation model is supported. Values of CFI and TLI > 0.9 together with SRMR and RMSEA < 0.08 indicate a supported mediation model (Hyland et al., 2014). The sample size was also calculated using the proposed mediation model. The mediation model contains 23 degrees of freedom, with the set‐up of type I error at 0.05, power at 0.9, null RMSEA at 0 and alternative RMSEA at 0.05, the desired sample size for the mediation model is around 480 (Preacher & Coffman, 2006). All statistical analyses were conducted using SPSS version 25 and Amos version 24.

## 4 RESULTS

A total of 516 emergency nurses with a mean age of 41.18 (*SD* = 8.24) years were used for this study. They were mostly females (*n* = 393; 76.16%), married (*n* = 407; 78.9%) with an average of 12.46 (*SD* = 10.35) years of working experience. These statistics is almost comparable to 2,500 nurses working in Qazvin province of whom 1,998 (79.92%) are females with a mean age of 37.56 years. Further details on the demographic information were shown in Table 1.

**TABLE 1:** Participants characteristics (*N* = 516)

	Mean ± *SD* or *n* (%)
Age (year)	41.18 ± 8.24
Gender (female)	393 (76.16%)
Years of experience	12.46 ± 10.35
<5	159 (30.8%)
5–9	107 (20.7%)
≥10	250 (48.4%)
Marital status	
Single	88 (17.1%)
Married	407 (78.9%)
Divorced/widowed	21 (4.1%)

Table 2 shows the Pearson *r* correlation matrix between fear of COVID‐19, job stress, medical professionals' burnout (emotional exhaustion, depersonalization and low sense of personal accomplishment) and mental health. All the correlation coefficients (*r* = 0.12–0.68) were significant (*p* < .05).

**TABLE 2:** Pearson's correlation matrix of the variables of interest

Variables	Fear of COVID−19	Job stress	EE	PA	DP	MCS
Fear of COVID−19^a^	—	0.269**	0.242**	−0.295**	0.231**	−0.271**
Job stress^b^		—	0.118**	−0.188**	0.125**	−0.477**
EE^c^			—	−0.534**	0.682**	−0.174**
PA^c^				—	−0.578*	0.194**
DP^c^					—	−0.121**
MCS^d^						—
Mean ± *SD*	21.45 ± 6.13	163.39 ± 68.80	4.30 ± 1.26	4.73 ± 1.29	2.24 ± 1.49	40.08 ± 30.60

Note

Abbreviations: DP, depersonalization; EE, emotional exhaustion; PA, personal accomplishment.

*p‐value < .05; **p‐value < .01.

^a^ Assessed using Fear of COVID‐19 Scale (FCV‐19S).

^b^ Assessed using Osipow occupational stress inventory.

^c^ Assessed using Maslach Burnout Inventory Human Services Survey for Medical Professionals (MBI‐HSS MP).

^d^ Assessed using the 12‐Item Short Form Health Survey (SF‐12).

Table 3 shows that there was a significant direct effect of fear of COVID‐19 on burnout (*β* = 0.311, 95% CI = 0.031–0.07), job stress (*β* = 2.69, 95% CI = 2.024–4.075) and mental health (*β* = 0.131, 95% CI = −1.101–−0.171); a significant direct effect of burnout on mental health (*β* = −0.80, 95% CI = −4.175–−0.667); and a significant direct effect of job stress on mental health (*β* = −0.430; 95% CI = −0.238–−0.161). Also, burnout (*β* = −0.085, 95% CI = −0.192–−0.054), job stress (*β* = −0.116, 95% CI = −0.844–−0.373) and job stress and burnout (sequential mediators; *β* = −0.140, 95% CI = −1.010–−0.472) were found to be significant mediators in the association between fear of COVID‐19 and mental health. In all, there was a significant total effect of fear of COVID‐19 on mental health of emergency nurses (*β* = −0.271, 95% CI = −1.801–−0.931). Moreover, the entire mediation model is supported by the satisfactory fit indices: χ2 = 70.307, *df* = 23; *p* < .001; CFI = 0.982, TLI = 0.961, SRMR = 0.0440, RMSEA = 0.064, 90% CI = [0.034, 0.095].

**TABLE 3:** Direct, indirect and total effects in the structural equation model testing relations among emergency nurses

Path	B (*SE*)	*β*	95% CI	
			LL	UL
Direct effects				
Fear of COVID−19*→*Burnout	0.051 (0.010)	0.311***	0.031	0.070
Fear of COVID−19*→*Job stress	2.970 (0.518)	0.269***	2.024	4.075
Fear of COVID−19*→*MCS	−0.652 (0.200)	−0.131***	−1.101	−0.171
Burnout*→*MCS	−2.421 (0.893)	−0.80**	−4.175	−0.667
Job stress*→*MCS	−0.194 (0.019)	−0.430***	−0.238	−0.161
Indirect effects				
Fear of COVID−19→ Burnout*→MCS*	−0.123 (0.035)	−0.085**	−0.192	−0.054
Fear of COVID−19→Job Stress*→*MCS	−0.576 (0.112)	−0.116***	−0.844	−0.373
Fear of COVID−19→Job Stress, Burnout →MCS	−0.699 (0.128)	−0.140***	−1.010	−0.472
Total effects				
Fear of COVID−19*→*MCS	−1.351 (0.220)	−0.271***	−1.801	−0.931

Age, sex, years of experience, and marital status were included as control variables in the structural equation model.

Abbreviations: B, unstandardized path coefficient; 95% CI, 95% confidence interval of unstandardized path coefficient; LL, lower limit of 95% CI; MCS, mental component summary; SE, standard error; *β*, standardized path coefficient; UL, upper limit of 95% CI.

*p < .05; ^**^ *p* < .01; ^***^ *p* < .001.

## 5 DISCUSSION

This cross‐sectional survey study examined the mediation roles of burnout and job stress in the association between fear of COVID‐19 and mental health. The correlation results revealed that fear of COVID‐19 related positively to job stress, emotional exhaustion and depersonalization but related negatively to personal accomplishment and mental health with small effect size for all these relationships (Cohen, 1988, 1992) which is similar to previous studies (Ahorsu, Imani, et al., 2020; Ahorsu, Lin, & Pakpour, 2020; Hu et al., 2020). This indicates that when emergency nurses' fear of COVID‐19 increases their stress at the workplace, emotional exhaustion and depersonalization symptoms may also increase and vice versa. However, when nurses' fear of COVID‐19 increases, their sense of personal accomplishment and mental health decreases and vice versa. The positive relationships between job stress, emotional exhaustion and depersonalization indicate that when nurses' stress on the job increases their burnout (emotional exhaustion and depersonalization) symptoms also increase (and vice versa). The negative relationships between job stress, sense of personal accomplishment and mental health indicate that when nurses' stress on the job increases, sense of personal accomplishment and mental health decrease and vice versa. The negative relationships between burnout (emotional exhaustion and depersonalization) symptoms and mental health indicate that the higher the nurses' experience burnout symptoms the poorer their mental health. The positive relationship between sense of personal accomplishment and mental health indicates that as nurses' sense of personal accomplishment increases their mental health also increase and vice versa. Similarly, previous studies have reported an association between fear of COVID‐19, mental health, burnout and stress (Ahorsu, Imani, et al., 2020; Ahorsu, Lin, & Pakpour, 2020; Hu et al., 2020).

These interrelationships (fear of COVID‐19, job stress, burnout and mental health) were further explained by the mediation analysis. The mediation results indicated that there were direct significant associations between (a) fear of COVID‐19 and mental health, (b) fear of COVID‐19 and mediators (job stress and burnout) and (c) mediators (job stress and burnout) and mental health. These findings strengthen the earlier relationships observed between the variables. That is, fear of an infectious disease such as COVID‐19 may significantly influence nurses' burnout, job stress or both which may influence their mental health as reported in previous studies (Ahorsu, Imani, et al., 2020; Ahorsu, Lin, & Pakpour, 2020; Hu et al., 2020; Shoja et al., 2020). Additionally, the mediating effects observed indicated an indirect effect of fear of COVID‐19 on mental health through burnout, job stress and job stress‐burnout with the strongest mediator effect being for the sequential job stress‐burnout, job stress and finally burnout. This indicates that fear of COVID‐19 indirectly influences the mental health of emergency nurses through job stress, burnout or both. In other words, fear of COVID‐19 significantly influenced emergency nurses' burnout and job stress positively which then influenced their mental health negatively. Hence, fear of COVID‐19, directly and indirectly, influences the mental health of emergency nurses.

These findings are very important as emergency nurses are mostly at the frontline of health services attending to all kinds of conditions including COVID‐19. This importance is against the backdrop that their burnout and compromised mental health situation may affect their job performance (e.g. quality of care, poor patient safety, adverse events, patient negative experience and medical errors) and, consequently, detrimental to their patients' life (Al Thobaity & Alshammari, 2020; De los Santos & Labrague, 2021; Labrague & de Los Santos, 2021; Shoja et al., 2020; Yayla & Eskici İlgin, 2021). Furthermore, the financial implications associated with the consequences of burnout and/or mental illness (absenteeism, sickness presenteeism, job performance, medical errors, turnover intention and job satisfaction) among nurses alone are significant enough to threaten the smooth running of the health service (Kline & Lewis, 2019; Medibank, 2011; National Taskforce for Humanity in Healthcare, 2018; Perry et al., 2015; Torjesen, 2018). This present study has provided significant evidence that fear of COVID‐19 may influence these negative events. Hence, hospital authorities (e.g. administrators and medical directors) should endeavour to provide adequate PPEs and other necessary resources needed to allay the fears of nurses in treating all kinds of patients during this COVID‐19 (or other infectious condition) pandemic period.

### 5.1 Limitation

This study had limitations. Only emergency nurses were used in this study; hence, the findings may be extended to nurses at the frontline of infectious diseases such as COVID‐19 but not necessarily to other departmental staffs in a hospital. Also, as different countries implemented different policies in managing COVID‐19, there may be the need replication of this study in order to holistically ascertain how the variables used in this study relate to other countries. This study used a cross‐sectional design which limits the cause and effect relationships between the variables and so readers should be cautious in overextending the findings of this study. In addition, self‐report measures were used in this study for gathering the data; hence, there may be biases such as social desirability. However, the use of appropriate administering procedures and the robustness of the psychometric properties for these measures suggest that the data are valid and trustworthy to an appreciable degree.

### 5.2 Conclusion

This cross‐sectional survey study revealed that fear of COVID‐19 may, directly and indirectly (through burnout, job stress or both), influence the mental health of emergency nurses negatively. This is a very important finding not only because of COVID‐19 but that it also pertains to the mental health of nurses and the life of patients they are treating. This finding suggests that fear of COVID‐19 and other fatal infectious condition for that matter influences the mental health of nurses. Hence, hospital authorities such as administrators and medical directors should provide enough resources to allay the fears of nurses in their line of duty during this COVID‐19 pandemic period. Replication of this study may be needed to ascertain how the variables used in this study differed from other countries.

## CONFLICT OF INTERESTS

None.

## ETHICAL STATEMENT

All participants provided written informed consent before study participation. The study protocol was approved by the Ethics Committee of Qazvin University of Medical Sciences (n. IR.QUMS.REC.1399.351) and conformed to the requirements of the Declaration of Helsinki. Approval from the participating institutions was also obtained. The data of the participants were kept confidential and used for academic research only.

## Data Availability

The authors elect not to share data. Research data are not shared for ethical and confidentiality reasons.

## References

[nop21154-bib-0001] Ahorsu, D. K. , Imani, V. , Lin, C.‐Y. , Timpka, T. , Broström, A. , Updegraff, J. A. , Årestedt, K. , Griffiths, M. D. , & Pakpour, A. H. (2020). Associations between fear of COVID‐19, mental health, and preventive behaviours across pregnant women and husbands: An actor‐partner interdependence modelling. International Journal of Mental Health and Addiction, 1–15. 10.1007/s11469-020-00340-x PMC7289236 32837427

[nop21154-bib-0002] Ahorsu, D. K. , Lin, C.‐Y. , Imani, V. , Saffari, M. , Griffiths, M. D. , & Pakpour, A. H. (2020). The Fear of COVID‐19 Scale: Development and Initial Validation. International Journal of Mental Health and Addiction, 1–9, 10.1007/s11469-020-00270-8 PMC7100496 32226353

[nop21154-bib-0003] Ahorsu, D. K. , Lin, C.‐Y. , & Pakpour, A. H. (2020). The association between health status and insomnia, mental health, and preventive behaviors: The mediating role of fear of COVID‐19. Gerontology and Geriatric Medicine, 6, 2333721420966081. 10.1177/2333721420966081 33195740 PMC7594224

[nop21154-bib-0004] Al Sabei, S. D. , Labrague, L. J. , Miner Ross, A. , Karkada, S. , Albashayreh, A. , Al Masroori, F. , & Al Hashmi, N. (2020). Nursing work environment, turnover intention, job burnout, and quality of care: The moderating role of job satisfaction. Journal of Nursing Scholarship, 52(1), 95–104. 10.1111/jnu.12528 31692251

[nop21154-bib-0005] Al Thobaity, A. , & Alshammari, F. (2020). Nurses on the Frontline against the COVID‐19 Pandemic: An Integrative Review. Dubai Medical Journal, 3(3), 87–92. 10.1159/000509361

[nop21154-bib-0006] Balanzá–Martínez, V. , Atienza–Carbonell, B. , Kapczinski, F. , & De Boni, R. B. (2020). Lifestyle behaviours during the COVID‐19–time to connect. Acta Psychiatrica Scandinavica, 141, 399–400. 10.1111/acps.13177 32324252 PMC7264786

[nop21154-bib-0007] Bentler, P. M. (1990). Comparative fit indexes in structural models. Psychological Bulletin, 107(2), 238–246. 10.1037/0033-2909.107.2.238 2320703

[nop21154-bib-0008] Çınar, D. , Kılıç Akça, N. , Zorba Bahçeli, P. , & Bağ, Y. (2021). Perceived stress and affecting factors related to COVID‐19 pandemic of emergency nurses in Turkey. Journal of Nursing Management, 29, 1916–1923. 10.1111/jonm.13329 33837591 PMC8250339

[nop21154-bib-0009] Cohen, J. (1988). Statistical power analysis for the behavioral sciences. Routledge.

[nop21154-bib-0010] Cohen, J. (1992). A power primer. Psychological Bulletin, 112(1), 155–159. 10.1037/0033-2909.112.1.155 19565683

[nop21154-bib-0011] Cui, S. , Jiang, Y. , Shi, Q. , Zhang, L. , Kong, D. , Qian, M. , & Chu, J. (2021). Impact of COVID‐19 on anxiety, stress, and coping styles in nurses in emergency departments and fever clinics: A cross‐sectional survey. Risk Management and Healthcare Policy, 14, 585–594. 10.2147/RMHP.S289782 33623449 PMC7894802

[nop21154-bib-0012] Dall’Ora, C. , Ball, J. , Reinius, M. , & Griffiths, P. (2020). Burnout in nursing: A theoretical review. Human Resources for Health, 18(1), 41. 10.1186/s12960-020-00469-9 32503559 PMC7273381

[nop21154-bib-0013] De los Santos, J. A. A. , & Labrague, L. J. (2021). The impact of fear of COVID‐19 on job stress, and turnover intentions of frontline nurses in the community: A cross‐sectional study in the Philippines. Traumatology, 27(1), 52–59. 10.1037/trm0000294

[nop21154-bib-0014] Fazeli, S. , Mohammadi Zeidi, I. , Lin, C.‐Y. , Namdar, P. , Griffiths, M. D. , Ahorsu, D. K. , & Pakpour, A. H. (2020). Depression, anxiety, and stress mediate the associations between internet gaming disorder, insomnia, and quality of life during the COVID‐19 outbreak. Addictive Behaviors Reports, 12, 100307. 10.1016/j.abrep.2020.100307 33110934 PMC7581367

[nop21154-bib-0015] Gössling, S. , Scott, D. , & Hall, C. M. (2020). Pandemics, tourism and global change: A rapid assessment of COVID‐19. Journal of Sustainable Tourism, 29(1), 1–20. 10.1080/09669582.2020.1758708

[nop21154-bib-0016] Gualano, M. R. , Sinigaglia, T. , Lo Moro, G. , Rousset, S. , Cremona, A. , Bert, F. , & Siliquini, R. (2021). The burden of burnout among healthcare professionals of intensive care units and emergency departments during the COVID‐19 pandemic: A systematic review. International Journal of Environmental Research and Public Health, 18(15), 8172. 10.3390/ijerph18158172 34360465 PMC8346023

[nop21154-bib-0017] Hamed, R. A. , Abd Elaziz, S. Y. , & Ahmed, A. S. (2020). Prevalence and predictors of burnout syndrome, post‐traumatic stress disorder, depression, and anxiety in nursing staff in various departments. Middle East Current Psychiatry, 27(1), 36. 10.1186/s43045-020-00044-x

[nop21154-bib-0018] Hu, D. , Kong, Y. , Li, W. , Han, Q. , Zhang, X. , Zhu, L. X. , Wan, S. W. , Liu, Z. , Shen, Q. U. , Yang, J. , He, H.‐G. , & Zhu, J. (2020). Frontline nurses' burnout, anxiety, depression, and fear statuses and their associated factors during the COVID‐19 outbreak in Wuhan, China: A large‐scale cross‐sectional study. EClinicalMedicine, 24, 100424. 10.1016/j.eclinm.2020.100424 32766539 PMC7320259

[nop21154-bib-0019] Hyland, P. , Shevlin, M. , Adamson, G. , & Boduszek, D. (2014). The organization of irrational beliefs in posttraumatic stress symptomology: Testing the predictions of REBT theory using structural equation modelling. Journal of Clinical Psychology, 70(1), 48–59. 10.1002/jclp.22009 23801553

[nop21154-bib-0020] Joreskog, K. , & Sorbom, D. (1981). LISREL V: Analysis of linear structural relationships hy the method of maximum likelihood. National Educational Resources.

[nop21154-bib-0021] Kline, R. , & Lewis, D. (2019). The price of fear: Estimating the financial cost of bullying and harassment to the NHS in England. Public Money & Management, 39(3), 166–174. 10.1080/09540962.2018.1535044

[nop21154-bib-0022] Kwon, C. Y. , Lee, B. , Kwon, O. J. , Kim, M. S. , Sim, K. L. , & Choi, Y. H. (2021). Emotional labor, burnout, medical error, and turnover intention among South Korean nursing staff in a University hospital setting. International Journal of Environmental Research and Public Health, 18(19), 10111. 10.3390/ijerph181910111 34639412 PMC8507784

[nop21154-bib-0023] Labrague, L. J. , & de Los Santos, J. A. A. (2021). Fear of Covid‐19, psychological distress, work satisfaction and turnover intention among frontline nurses. Journal of Nursing Management, 29(3), 395–403. 10.1111/jonm.13168 32985046 PMC7537256

[nop21154-bib-0024] Lippi, G. , Henry, B. M. , & Sanchis‐Gomar, F. (2020). Physical inactivity and cardiovascular disease at the time of coronavirus disease 2019 (COVID‐19). European Journal of Preventive Cardiology, 27(9), 906–908. 10.1177/2047487320916823 32270698 PMC7717305

[nop21154-bib-0025] Maslach, C. , Jackson, S. , & Leiter, M. (2018). Maslach burnout inventory manual, 4th ed. Mind Garden Inc.

[nop21154-bib-0026] Medibank . (2011). Sick at Work: The cost of presenteeism to your business and the economy. In Economic Modelling of the Cost of Presenteeism in Australia: 2011 Update (Medibank, KPMG Econtech). http://www.medibank.com.au/Client/Documents/Pdfs/sick_at_work.pdf

[nop21154-bib-0027] Moalemi, S. , Kavosi, Z. , Beygi, N. , Deghan, A. , Karimi, A. , & Parvizi, M. M. (2018). Evaluation of the Persian Version of Maslach Burnout Inventory‐Human Services Survey among Iranian nurses: Validity and reliability. Galen Medical Journal, 7, e995. 10.22086/gmj.v0i0.995 34466422 PMC8343696

[nop21154-bib-0028] Montazeri, A. , Vahdaninia, M. , Mousavi, S. J. , Asadi‐Lari, M. , Omidvari, S. , & Tavousi, M. (2011). The 12‐item medical outcomes study short form health survey version 2.0 (SF‐12v2): A population‐based validation study from Tehran, Iran. Health and Quality of Life Outcomes, 9, 12. 10.1186/1477-7525-9-12 21385359 PMC3063185

[nop21154-bib-0029] National Taskforce for Humanity in Healthcare (2018). The business case for humanity in healthcare. https://www.vocera.com/public/pdf/NTHBusinessCase_final003.pdf

[nop21154-bib-0030] Nicola, M. , Alsafi, Z. , Sohrabi, C. , Kerwan, A. , Al‐Jabir, A. , Iosifidis, C. , Agha, M. , & Agha, R. (2020). The socio‐economic implications of the coronavirus pandemic (COVID‐19): A review. International Journal of Surgery, 78, 185–193. 10.1016/j.ijsu.2020.04.018 32305533 PMC7162753

[nop21154-bib-0031] Osipow, S. H. (1998). Occupational Stress Inventory‐Revised Edition (OSI‐R): Professional manual. Psychological Assessment Resources Inc.

[nop21154-bib-0032] Pakpour, A. H. , Nourozi, S. , Molsted, S. , Harrison, A. P. , Nourozi, K. , & Fridlund, B. (2011). Validity and reliability of short form‐12 questionnaire in Iranian hemodialysis patients. Iranian Journal of Kidney Disease, 5(3), 175–181. 21525577

[nop21154-bib-0033] Perry, L. , Lamont, S. , Brunero, S. , Gallagher, R. , & Duffield, C. (2015). The mental health of nurses in acute teaching hospital settings: A cross‐sectional survey. BMC Nursing, 14(1), 1–8. 10.1186/s12912-015-0068-8 25904820 PMC4405850

[nop21154-bib-0034] Preacher, K. J. , & Coffman, D. L. (2006). Computing power and minimum sample size for RMSEA [Computer software]. http://quantpsy.org/

[nop21154-bib-0035] Rajabimajd, N. , Alimoradi, Z. , & Griffiths, M. D. (2021). Impact of COVID‐19‐related fear and anxiety on job attributes: A systematic review. Asian Journal of Social Health and Behavior, 4(2), 51–55. 10.4103/shb.shb_24_21

[nop21154-bib-0036] Sharifian, S. A. , Aminian, O. , Kiyani, M. , Barouni, S. H. , & Amiri, F. (2006). The evaluation of the degree of occupational stress and factors influencing it in forensic physicians working in Legal Medicine Organization in Tehran ‐ Autumn of 2005. Scientific Journal of Forensic Medicine, 12(3), 144–150.

[nop21154-bib-0037] Shoja, E. , Aghamohammadi, V. , Bazyar, H. , Moghaddam, H. R. , Nasiri, K. , Dashti, M. , Choupani, A. , Garaee, M. , Aliasgharzadeh, S. , & Asgari, A. (2020). Covid‐19 effects on the workload of Iranian healthcare workers. BMC Public Health, 20(1), 1636. 10.1186/s12889-020-09743-w 33138798 PMC7605333

[nop21154-bib-0038] Sung, C.‐W. , Lu, T.‐C. , Fang, C.‐C. , Huang, C.‐H. , Chen, W.‐J. , Chen, S.‐C. , & Tsai, C.‐L. (2020). Impact of COVID‐19 pandemic on emergency department services acuity and possible collateral damage. Resuscitation, 153, 185–186. 10.1016/j.resuscitation.2020.06.021 32585231 PMC7308005

[nop21154-bib-0039] Torjesen, I. (2018). Bullying and harassment cost NHS over £2bn a year, study suggests. BMJ, 363, k4463. 10.1136/bmj.k4463 30355582

[nop21154-bib-0040] Tucker, L. R. , & Lewis, C. (1973). A reliability coefficient for maximum likelihood factor analysis. Psychometrika, 38(1), 1–10. 10.1007/BF02291170

[nop21154-bib-0041] Van Lancker, W. , & Parolin, Z. (2020). COVID‐19, school closures, and child poverty: A social crisis in the making. The Lancet Public Health, 5(5), e243–e244. 10.1016/S2468-2667(20)30084-0 32275858 PMC7141480

[nop21154-bib-0042] Wang, J. , Zong, L. , Zhang, J. , Sun, H. , Harold Walline, J. , Sun, P. , Xu, S. , Li, Y. , Wang, C. , Liu, J. , Li, F. , Xu, J. , Li, Y. I. , Yu, X. , & Zhu, H. (2020). Identifying the effects of an upgraded ‘fever clinic’ on COVID‐19 control and the workload of emergency department: Retrospective study in a tertiary hospital in China. British Medical Journal Open, 10(8), e039177. 10.1136/bmjopen-2020-039177 PMC7440187 32819955

[nop21154-bib-0043] Ware, J. E. Jr , Kosinski, M. , & Keller, S. D. (1996). A 12‐Item Short‐Form Health Survey: Construction of scales and preliminary tests of reliability and validity. Medical Care, 34(3), 220–233. 10.1097/00005650-199603000-00003 8628042

[nop21154-bib-0044] Wiersinga, W. J. , Rhodes, A. , Cheng, A. C. , Peacock, S. J. , & Prescott, H. C. (2020). Pathophysiology, transmission, diagnosis, and treatment of Coronavirus disease 2019 (COVID‐19): A review. JAMA, 324(8), 782–793. 10.1001/jama.2020.12839 32648899

[nop21154-bib-0045] World Health Organisation . (2021). COVID‐19 Weekly Epidemiological Update on COVID‐19—5 October 2021. https://www.who.int/publications/m/item/weekly‐epidemiological‐update‐on‐covid‐19‐‐‐5‐october‐2021

[nop21154-bib-0046] Zare, S. , Kazemi, R. , Izadi, A. , & Smith, A. (2021). Beyond the outbreak of COVID‐19: Factors affecting burnout in nurses in Iran. Annals of Global Health, 87(1), 51. 10.5334/aogh.3190 34221904 PMC8231475

[nop21154-bib-0049] Yayla, A. , & Eskici İlgin, V. (2021). The relationship of nurses' psychological well‐being with their coronaphobia and work‐life balance during the COVID‐19 pandemic: A cross‐sectional study. Journal of clinical nursing, 30(21‐22), 3153–3162. 10.1111/jocn.15783 34337812 PMC8447145

[nop21154-bib-0047] Zeng, L.‐N. , Zhang, J.‐W. , Zong, Q.‐Q. , Chan, S.‐C. , Browne, G. , Ungvari, G. S. , Chen, L.‐G. , & Xiang, Y.‐T. (2020). Prevalence of burnout in mental health nurses in China: A meta‐analysis of observational studies. Archives of Psychiatric Nursing, 34(3), 141–148. 10.1016/j.apnu.2020.03.006 32513464

[nop21154-bib-0048] Zhan, Y. , Ma, S. , Jian, X. , Cao, Y. , & Zhan, X. (2020). The current situation and influencing factors of job stress among frontline nurses assisting in Wuhan in fighting COVID‐19. Frontiers in Public Health, 8, 579866. 10.3389/fpubh.2020.579866 33194981 PMC7649821

